# Supplementary material for: Changes in cortisol awakening responses (CAR) in menopausal women through short-term marine healing retreat program with specific factors affecting each CAR index
Source: PLoS One. 2023 Apr 19;18(4):e0284627. doi: 10.1371/journal.pone.0284627 (PMC10115294; doi:10.1371/journal.pone.0284627)
Supplement: S1 Table — Each bar represents the mean ± SD. p-values were obtained by ANOVA. (DOCX) [file pone.0284627.s001.docx]

Table S1. Differences in Sleep Efficiency based on age group before the marine healing program

| **Age group** | **n** | **Sleep Efficiency %** | **p** |
| --- | --- | --- | --- |
| **Age** ≤55 | 14 | 94.7 ± 2.7 | 0.069 |
| 55~60 | 21 | 92.0 ± 4.9 |  |
| >60 | 16 | 90.9 ± 4.9 |  |

Each bar represents the mean ± SD. p-values were obtained by ANOVA.
