# Supplementary material for: Changes in cortisol awakening responses (CAR) in menopausal women through short-term marine healing retreat program with specific factors affecting each CAR index
Source: PLoS One. 2023 Apr 19;18(4):e0284627. doi: 10.1371/journal.pone.0284627 (PMC10115294; doi:10.1371/journal.pone.0284627)
Supplement: S2 Table — R2 = 0.23 Adjusted R2 = 0.16 p = 0.019*. p-values were obtained by multivariate regression analysis. *p-value<0.05. (DOCX) [file pone.0284627.s002.docx]

Table S2. Factors affecting AVE before the marine healing program through multivariate regression analysis

| **Variable** | **B** | **Standard**  **Error** | **t** | **p** |
| --- | --- | --- | --- | --- |
| Age | -0.322 | 41.0 | -1.14 | 0.26 |
| BMI | -0.696 | 0.282 | -1.30 | 0.20 |
| LF/HF ratio | -0.728 | 0.535 | -1.18 | 0.21 |
| Sleep Efficiency % | 0.710 | 0.617 | 2.19 | 0.03^*^ |
| R2=0.25 Adjusted R2=0.18 p=0.012^*^. p-values were obtained by multivariate regression analysis. *p-value<0.05. | | | | |
