# Supplementary material for: Changes in cortisol awakening responses (CAR) in menopausal women through short-term marine healing retreat program with specific factors affecting each CAR index
Source: PLoS One. 2023 Apr 19;18(4):e0284627. doi: 10.1371/journal.pone.0284627 (PMC10115294; doi:10.1371/journal.pone.0284627)
Supplement: S3 Table — R2 = 0.16 Adjusted R2 = 0.09 p = 0.06. p-values were obtained by multivariate regression analysis. (DOCX) [file pone.0284627.s003.docx]

Table S3. Factors affecting AUCg before the marine healing program through multivariate regression analysis

| **Variable** | **B** | **Standard**  **Error** | **t** | **p** |
| --- | --- | --- | --- | --- |
| Age | -15.73 | 13.65 | -1.15 | 0.25 |
| BMI | -31.69 | 25.84 | -1.23 | 0.22 |
| LF/HF ratio | -33.49 | 29.78 | -1.12 | 0.26 |
| Sleep Efficiency % | 32.12 | 15.65 | 2.05 | 0.04^*^ |
| R2=0.23 Adjusted R2=0.16 p=0.019^*^. p-values were obtained by multivariate regression analysis. *p-value<0.05. | | | | |
