# Supplementary material for: Changes in cortisol awakening responses (CAR) in menopausal women through short-term marine healing retreat program with specific factors affecting each CAR index
Source: PLoS One. 2023 Apr 19;18(4):e0284627. doi: 10.1371/journal.pone.0284627 (PMC10115294; doi:10.1371/journal.pone.0284627)
Supplement: S4 Table — R2 = 0.07 Adjusted R2 = -0.01 p = 0.50. p-values were obtained by multivariate regression analysis. (DOCX) [file pone.0284627.s004.docx]

**Table S4.** Factors affecting AUCi before the marine healing program through multivariate regression analysis

| **Variable** | **B** | **Standard**  **Error** | **t** | **p** |
| --- | --- | --- | --- | --- |
| Age | -32.15 | 12.37 | -2.60 | 0.01 |
| LF/HF ratio | -20.20 | 26.82 | -0.75 | 0.46 |
| Sleep Efficiency % | 2.89 | 13.99 | 0.21 | 0.84 |
| R2=0.16 Adjusted R2=0.09 p=0.06. p-values were obtained by multivariate regression analysis. | | | | |
