# Supplementary material for: Changes in cortisol awakening responses (CAR) in menopausal women through short-term marine healing retreat program with specific factors affecting each CAR index
Source: PLoS One. 2023 Apr 19;18(4):e0284627. doi: 10.1371/journal.pone.0284627 (PMC10115294; doi:10.1371/journal.pone.0284627)
Supplement: S5 Table — R2 = 0.10 Adjusted R2 = 0.02 p = 0.30. p-values were obtained by multivariate regression analysis. (DOCX) [file pone.0284627.s005.docx]

**Table S5.** Factors affecting AVE after the marine healing program through multivariate regression analysis

| **Variable** | **B** | **Standard**  **Error** | **t** | **p** |
| --- | --- | --- | --- | --- |
| Age | -0.24 | 0.26 | -0.92 | 0.36 |
| BMI | 0.42 | 0.46 | 0.92 | 0.36 |
| LF/HF ratio | -0.92 | 1.13 | -0.82 | 0.41 |
| Sleep Efficiency % | -0.29 | 0.27 | -1.07 | 0.29 |
| R2=0.07 Adjusted R2=-0.01 p=0.50. p-values were obtained by multivariate regression analysis. | | | | |
