# Supplementary material for: Changes in cortisol awakening responses (CAR) in menopausal women through short-term marine healing retreat program with specific factors affecting each CAR index
Source: PLoS One. 2023 Apr 19;18(4):e0284627. doi: 10.1371/journal.pone.0284627 (PMC10115294; doi:10.1371/journal.pone.0284627)
Supplement: S6 Table — R2 = 0.03 Adjusted R2 = -0.05 p = 0.82. p-values were obtained by multivariate regression analysis. (DOCX) [file pone.0284627.s006.docx]

**Table S6.** Factors affecting AUCg after the marine healing program through multivariate regression analysis

| **Variable** | **B** | **Standard**  **Error** | **t** | **p** |
| --- | --- | --- | --- | --- |
| Age | -17.48 | 12.62 | -1.39 | 0.17 |
| BMI | 24.11 | 22.81 | 1.06 | 0.3 |
| LF/HF ratio | -53.31 | 55.71 | -0.96 | 0.34 |
| Sleep Efficiency % | -14.23 | 13.41 | -1.06 | 0.29 |
| R2=0.10 Adjusted R2=0.02 p=0.30. p-values were obtained by multivariate regression analysis. | | | | |
