# Supplementary material for: Changes in cortisol awakening responses (CAR) in menopausal women through short-term marine healing retreat program with specific factors affecting each CAR index
Source: PLoS One. 2023 Apr 19;18(4):e0284627. doi: 10.1371/journal.pone.0284627 (PMC10115294; doi:10.1371/journal.pone.0284627)
Supplement: S7 Table — R2 = 0.22 Adjusted R2 = 0.16 p = 0.017*. p-values were obtained by multivariate regression analysis. *p-value<0.05;**p < 0.01. (DOCX) [file pone.0284627.s007.docx]

**Table S7.** Factors affecting AUCi after the marine healing program through multivariate regression analysis

| **Variable** | **B** | **Standard**  **Error** | **t** | **p** |
| --- | --- | --- | --- | --- |
| Age | 0.12 | 12.31 | 0.01 | 0.99 |
| BMI | -2.05 | 22.25 | -0.09 | 0.93 |
| LF/HF ratio | -61.7 | 54.34 | -1.14 | 0.26 |
| Sleep Efficiency % | -4.88 | 13.09 | -0.37 | 0.71 |
| R2=0.03 Adjusted R2=-0.05 p=0.82. p-values were obtained by multivariate regression analysis. | | | | |
