# Supplementary material for: Changes in cortisol awakening responses (CAR) in menopausal women through short-term marine healing retreat program with specific factors affecting each CAR index
Source: PLoS One. 2023 Apr 19;18(4):e0284627. doi: 10.1371/journal.pone.0284627 (PMC10115294; doi:10.1371/journal.pone.0284627)
Supplement: S8 Table — R2 = 0.19 Adjusted R2 = 0.13 p = 0.028*. p-values were obtained by multivariate regression analysis. *p-value<0.05;**p < 0.01. (DOCX) [file pone.0284627.s008.docx]

**Table S8.** Factors affecting changes in AVE through the marine healing program through multivariate regression analysis

| **Variable** | **B** | **Standard**  **Error** | **t** | **p** |
| --- | --- | --- | --- | --- |
| BMI | 1.36 | 0.43 | 3.19 | 0.002^**^ |
| LF/HF ratio | 0.05 | 0.49 | 0.09 | 0.93 |
| Sleep Efficiency % | -0.09 | 0.26 | -0.36 | 0.72 |
| R2=0.22 Adjusted R2=0.16 p=0.017^*^. p-values were obtained by multivariate regression analysis. *p-value<0.05;**p < 0.01. | | | | |
