# Supplementary material for: Changes in cortisol awakening responses (CAR) in menopausal women through short-term marine healing retreat program with specific factors affecting each CAR index
Source: PLoS One. 2023 Apr 19;18(4):e0284627. doi: 10.1371/journal.pone.0284627 (PMC10115294; doi:10.1371/journal.pone.0284627)
Supplement: S9 Table — R2 = 0.14 Adjusted R2 = 0.08 p = 0.09. p-values were obtained by multivariate regression analysis. (DOCX) [file pone.0284627.s009.docx]

**Table S9.** Factors affecting changes in AUCg through the marine healing program through multivariate regression analysis

| **Variable** | **B** | **Standard**  **Error** | **t** | **p** |
| --- | --- | --- | --- | --- |
| BMI | 64.92 | 21.51 | 3.02 | 0.004^**^ |
| LF/HF ratio | -0.8 | 24.54 | -0.03 | 0.97 |
| Sleep Efficiency % | -2.67 | 13.04 | -0.21 | 0.84 |
| R2=0.19 Adjusted R2=0.13 p=0.028^*^. p-values were obtained by multivariate regression analysis. *p-value<0.05;**p < 0.01. | | | | |
