# Supplementary material for: Changes in cortisol awakening responses (CAR) in menopausal women through short-term marine healing retreat program with specific factors affecting each CAR index
Source: PLoS One. 2023 Apr 19;18(4):e0284627. doi: 10.1371/journal.pone.0284627 (PMC10115294; doi:10.1371/journal.pone.0284627)
Supplement: S10 Table — R2 = 0.25 Adjusted R2 = 0.18 p = 0.012*. p-values were obtained by multivariate regression analysis. *p-value<0.05. (DOCX) [file pone.0284627.s010.docx]

**Table S10.** Factors affecting changes in AUCi through the marine healing program through multivariate regression analysis

| **Variable** | **B** | **Standard**  **Error** | **t** | **p** |
| --- | --- | --- | --- | --- |
| Age | 28.89 | 12.88 | 2.24 | 0.03 |
| LF/HF ratio | -27.17 | 27.85 | -0.98 | 0.33 |
| Sleep Efficiency % | 9.89 | 14.62 | 0.68 | 0.5 |
| R2=0.14 Adjusted R2=0.08 p=0.09. p-values were obtained by multivariate regression analysis. | | | | |
