# Supplementary material for: Changes in cortisol awakening responses (CAR) in menopausal women through short-term marine healing retreat program with specific factors affecting each CAR index
Source: PLoS One. 2023 Apr 19;18(4):e0284627. doi: 10.1371/journal.pone.0284627 (PMC10115294; doi:10.1371/journal.pone.0284627)
Supplement: S11 Table — Each bar represents the mean ± SD. p-values were obtained by student t-test. (DOCX) [file pone.0284627.s011.docx]

**Table S11.** Differences in the exercise intensity depending on BMI groups in the marine healing program

| **BMI group** | **n** | **Exercise intensity** | **p** |
| --- | --- | --- | --- |
| Normal & Overweight (<25) | 27 | 76.7 ± 12.6 | 0.436 |
| Obese (25~) | 24 | 79.6 ± 13.7 |  |

Each bar represents the mean ± SD. p-values were obtained by student t-test.
