# Supplementary material for: Changes in cortisol awakening responses (CAR) in menopausal women through short-term marine healing retreat program with specific factors affecting each CAR index
Source: PLoS One. 2023 Apr 19;18(4):e0284627. doi: 10.1371/journal.pone.0284627 (PMC10115294; doi:10.1371/journal.pone.0284627)
Supplement: S12 Table — Each bar represents the mean ± SD. p-values were obtained by student t-test. *p-value<0.05;**p < 0.01. TG: triglyceride; BDNF: brain-derived neurotrophic factor. (DOCX) [file pone.0284627.s012.docx]

**Table S12.** Differences in the changes of TG and BDNF depending on BMI groups through the marine healing program

| **BMI group** | **n** | **Change of TG**  **(mg/dL)** | **p** | **Change of BDNF**  **(pg/ml)** | **p** |
| --- | --- | --- | --- | --- | --- |
| Normal & Overweight (<25) | 27 | -42.9 ± 31.7 | 0.007^**^ | -6134 ± 9958 | 0.04^*^ |
| Obese (25~) | 24 | -84 ± 69.5 |  | -13161 ± 13322 |  |

Each bar represents the mean ± SD. p-values were obtained by student t-test. *p-value<0.05;**p < 0.01. TG : triglyceride; BDNF : brain-derived neurotrophic factor.
