# Supplementary material for: Changes in cortisol awakening responses (CAR) in menopausal women through short-term marine healing retreat program with specific factors affecting each CAR index
Source: PLoS One. 2023 Apr 19;18(4):e0284627. doi: 10.1371/journal.pone.0284627 (PMC10115294; doi:10.1371/journal.pone.0284627)
Supplement: Attachment — Submitted filename: 230117_Response to reviewers.docx [file pone.0284627.s013.docx]

**Jan 17, 2023**

**Title: Changes in cortisol awakening responses (CAR) in menopausal women through short-term marine healing retreat program with specific factors affecting each CAR parameter**

**Dear Dr. Juan-Luis Castillo-Navarrete:**

We really appreciate the reviewer’s comments and found that they helped to improve our paper. We have revised the manuscript with tables responded to the reviewers’ advices, and the responses to reviewer’s comments are as follows;

**Responses to reviewer comments:** The reviewer’s advice has been bold letters and our response follows. In addition, the contents reflected in the manuscript were written in red.

**Comments to the Author**

**Reviewer #1:**

Negative answer to the question “the manuscript presented in an intelligible fashion and written in standard English?”

We admit that there were deficiencies in our writing using standard English. We have written again based on the reviewer’s comments and the manuscript was edited again by professional English proofreading service.

(1) **With all due respect for the work of the authors, it is however my opinion that the MS, who deals with an important topic in the field of improving the quality of life, needs a profound reorganization. The introduction is unnecessarily verbose. It does not stimulate or justify interest in the topic right from the first sentences.**

Thank you very much for your thoughtful comments. We re-organized the introduction to focus on the goal of the research. And the first sentence of the introduction was also modified to highlight the relationship between CAR and health risk.

[Introduction] (p3, L41~p4, L78)

The changed parts of the introduction are most sentences of the introduction because the total contents of introduction were re-organized. Please see the introduction of the revised manuscript.

(2) **The purpose of the research is not clear, nor the choice of the population is justified (the experimental group is not at all described in its clinical characteristics in the relative missing paragraph "study population", for example for the possible intake of drugs that could modify HPA axis activities; spontaneous/surgical menopause?)**

We greatly appreciate the comments. Based on the reviewer's opinion, the introduction was revised as the response to number 1 comment. And the sentences were modified to clearly express the purpose of the study (last paragraph of introduction).

And we added information about “study population” status in the recruitment of subject section in Materials and Methods to justify the choice for the study subjects, for example, their medications and surgical experiences, and menopausal status.

[Introduction] (p4, L73~L78)

Therefore, this study aimed to identify the major factors affecting the CAR, excluding stress factors, in menopausal women. We evaluated the effects of various factors such as age, obesity, sleep, and circadian rhythm on the CAR in menopausal women who participated in a marine healing retreat program, including beach yoga and Nordic walking. We found that CAR was associated with sleep efficiency before the program, and a change in CAR was associated with obesity throughout the exercise retreat program.

[Materials and Methods] (p5, L81~L85)

The participants were recruited in three batches of 20 from February to June 2021. A total of 60 female participants in their 50s and 60s who were non-smokers and had no difficulty of exercising were recruited. The participants had never undergone menopause-related surgery and were in spontaneous menopause. Additionally, the participants were not taking any medications known to affect HPA axis function, including opiates, steroids, or antipsychotics associated with cortisol secretion (18).

(3) **The results paragraph should be rewritten by providing the reader with a clear and detailed explanation of what has resulted, and taking away the feeling of simply being sent back to reading what is reported in the table!**

We greatly appreciate the comments. Based on the reviewer's comments, we have changed the result paragraph to focus on the main results to give the readers clear and detailed explanation of the results. Also, tables S1-S3 in the previous submitted version were deleted because those tables repeated the results and did not have important meaning, and thank you for your understanding in advance that the supplementary table numbers were finally modified**.**

[Results] (p9, L160~ p10, L197)

Because the many sentences of results were re-organized or re-written, we could not show the whole changed parts of the results. Please see the results of the revised manuscript.

(4) **Discussion may be improved including possible mechanisms and including a comment/comparison with similar studies in the area.**

We greatly appreciate the comments. Our main findings identified significant changes of CAR in obese subjects through an exercise program, and we added a related discussion. We proposed possible mechanisms such as the associations of oxidative stress and CAR with BDNF for obese subjects to improve the CAR. And our proposed mechanisms also compared with previous similar studies.

**[**Discussion] (p12, L246~ p13, L261)

There was no significant change in the BMI in obese and non-obese participants throughout the program, and there were no differences in exercise intensity based on heart rate measurements during the program (Table S11). However, the TG and BDNF levels significantly decreased in the obese group after the program (p<0.05; Table S12). Aerobic exercises reduce the TGs in adults who are overweight or have obesity (45), and exercising and dietary control have been shown to reduce BDNF in women with obesity (46). In addition, a study has reported that the recognition of bodyweight-associated stigma increases oxidative stress and activates the HPA axis, which affects the CAR (47). While physical activity promotes health benefits, strenuous or prolonged exercise increases the production of reactive oxygen species in the blood and skeletal muscles (48). Previous studies have reported that the circulating BDNF can act as a protective agent against oxidative damage; as oxidative stress increases and the BDNF levels decrease (49, 50). Our study has the limitation of not directly investigating changes in oxidative stress. However, we hypothesize that the exercise program employed in this study may have reduced the circulating BDNF levels against increased oxidative stress in participants with obesity. Thus, the retreat program seems to be effective in promoting changes of the CAR parameters associated with changes of BDNF in obese subjects.

**Reviewer #2:**

(1) **Congratulations to the authors for make a detailed analysis of multiple variables that may affect changes in cortisol awakening responses (CAR) in menopausal women through short-term marine healing retreat program.**

We thank the reviewer who evaluated the research value of the paper.

(2) **Statistical analysis is adequate for the study of the data available. It is necessary to review the paragraph that describes Table 3 in the results. The multivariate analysis of WHR on AUCi parameter is not found in supplementary table S9.**

We greatly appreciate the comments. As the reviewer commented, we found the description for table S9 was insufficient. The WHR variable was excluded because WHR was not a significant factor when performing multivariate analysis. Therefore, it is correct that there is no WHR in table S9. So, we have amended related sentences not to be misleading.

**[**Results] (p9, L178~ p10, L183)

There were no significant factors associated with CAR parameters observed after the program. The participants’ age, BMI, blood pressure, female hormone levels, and medical history were analyzed to investigate differences in AVE, AUCg, and AUCi after the program (Table 3). The results showed that AUCi increased significantly more in the group with a normal waist-to-hip ratio (WHR) than in the group with a high WHR (p=0.02). However, in multivariate analysis, no factors significantly affected AUCi (Tables S4-S6).

(3) **In the same paragraph, the description of table S2 that indicates the existence of significant differences between BMI values for the high, middle, and low AUCi groups is confusing, because the values 25±2.6; 24±2.5 and 25±2.7 for high, middle and low AUCi respectively, are very similar.**

We appreciate the reviewer's thoughtful comments. We found the errors of the table, and this table showed the similar results for the association of BMI and CAR in Table 2. Therefore, we deleted the table and corresponding statements.

**[**Results] (p9, L170~L172)

The CAR parameters showed variable results. The high sleep efficiency group showed higher AVE and AUCg among the CAR parameters (p=0.02, p=0.01). AUCi did not reveal a significant difference between the high and low sleep efficiency groups (Table 2). ~~BMI values were significantly different among the high, middle, and low AUCi groups; however, the mid-level AUCi group had the lowest BMI (p=0.03); thus, the relationship between the BMI group and AUCi did not show a dose-response relationship (Table S2).~~

(4) **The authors present a comprehensive discussion of their results associated with the main findings of the study. Based on the changes in AVE and AUCg evidenced according to the BMI, it would be interesting for subsequent studies with larger populations to include the analysis of changes in oxidative stress markers.**

We greatly appreciate the comments, especially the comment about oxidative stress. Based on the reviewer's comments, we added a discussion that could explain the difference in CAR changes according to the BMI group in relation to oxidative stress (L251-261 of the discussion). Thanks for comments again that improves the discussion.

[Discussion] (p13, L251~ L261)

In addition, a study has reported that the recognition of bodyweight-associated stigma increases oxidative stress and activates the HPA axis, which affects the CAR (47). While physical activity promotes health benefits, strenuous or prolonged exercise increases the production of reactive oxygen species in the blood and skeletal muscles (48). Previous studies have reported that the circulating BDNF can act as a protective agent against oxidative damage; as oxidative stress increases and the BDNF levels decrease (49, 50). Our study has the limitation of not directly investigating changes in oxidative stress. However, we hypothesize that the exercise program employed in this study may have reduced the circulating BDNF levels against increased oxidative stress in participants with obesity. Thus, the retreat program seems to be effective in promoting changes of the CAR parameters associated with changes of BDNF in obese subjects.

**References**

1.	Wilhelm I, Born J, Kudielka BM, Schlotz W, Wust S. Is the cortisol awakening rise a response to awakening? Psychoneuroendocrinology. 2007;32(4):358-66.

2.	Sephton SE, Sapolsky RM, Kraemer HC, Spiegel D. Diurnal cortisol rhythm as a predictor of breast cancer survival. Journal of the National Cancer Institute. 2000;92(12):994-1000.

3.	Sauer T, Tottenham LS, Ethier A, Gordon JL. Perimenopausal vasomotor symptoms and the cortisol awakening response. Menopause. 2020;27(11):1322-7.

4.	Deuschle M, Gotthardt U, Schweiger U, Weber B, Körner A, Schmider J, et al. With aging in humans the activity of the hypothalamus-pituitary-adrenal system increases and its diurnal amplitude flattens. Life sciences. 1997;61(22):2239-46.

5.	Oosterholt BG, Maes JHR, Van der Linden D, Verbraak MJPM, Kompier MAJ. Burnout and cortisol: Evidence for a lower cortisol awakening response in both clinical and non-clinical burnout. Journal of Psychosomatic Research. 2015;78(5):445-51.

6.	Budde H, Akko DP, Ainamani HE, Murillo-Rodríguez E, Weierstall R. The impact of an exercise training intervention on cortisol levels and post-traumatic stress disorder in juveniles from an Ugandan refugee settlement: study protocol for a randomized control trial. Trials. 2018;19(1):364.

18.	Locatelli V, Bresciani E, Tamiazzo L, Torsello A. Central nervous system-acting drugs influencing hypothalamic-pituitary-adrenal axis function. Endocr Dev. 2010;17:108-20.

45.	Kelley GA, Kelley KS, Vu Tran Z. Aerobic exercise, lipids and lipoproteins in overweight and obese adults: a meta-analysis of randomized controlled trials. Int J Obes (Lond). 2005;29(8):881-93.

46.	Glud M, Christiansen T, Larsen LH, Richelsen B, Bruun JM. Changes in Circulating BDNF in relation to Sex, Diet, and Exercise: A 12-Week Randomized Controlled Study in Overweight and Obese Participants. Journal of Obesity. 2019;2019:4537274.

47.	Tomiyama AJ, Epel ES, McClatchey TM, Poelke G, Kemeny ME, McCoy SK, et al. Associations of weight stigma with cortisol and oxidative stress independent of adiposity. Health Psychol. 2014;33(8):862-7.

48.	Powers SK, Jackson MJ. Exercise-induced oxidative stress: cellular mechanisms and impact on muscle force production. Physiological reviews. 2008;88(4):1243-76.

49.	Valvassori SS, Arent CO, Steckert AV, Varela RB, Jornada LK, Tonin PT, et al. Intracerebral administration of BDNF protects rat brain against oxidative stress induced by ouabain in an animal model of mania. Molecular neurobiology. 2015;52(1):353-62.

50.	He T, Katusic ZS. Brain-derived neurotrophic factor increases expression of MnSOD in human circulating angiogenic cells. Microvascular research. 2012;83(3):366-71.

**Responses to Journal requirements:**

**1. Please ensure that your manuscript meets PLOS ONE's style requirements, including those for file naming.**

We formatted to meet PLOS ONE’s style requirements. Thank you for your review.

**2. Please provide additional details regarding participant consent. In the ethics statement in the Methods and online submission information, please ensure that you have specified what type you obtained (for instance, written or verbal, and if verbal, how it was documented and witnessed**

We added additional details regarding participant consent. we have amended these statements in the Methods section of the manuscript, and added the same text to the “Ethics Statement” field of the submission form.

[Materials and Methods] (p5, L88~ L90)

The study procedures were approved by the institutional review board (IRB) of Korea University (KUIRB-2021-0094-06). Detailed objectives and procedures of the study were informed, and written consent was obtained from the participants.

**3. We note that the grant information you provided in the ‘Funding Information’ and ‘Financial Disclosure’ sections do not match. When you resubmit, please ensure that you provide the correct grant numbers for the awards you received for your study in the ‘Funding Information’ section.**

We received funding for this study from the Wando Marine Healing Blue Zone Creation Project funded by the Presidential Committee for Balanced National Development in South Korea, but it has no grant number. Therefore, please understand us for not being able to provide a grant number.

**4. Thank you for stating the following in the Acknowledgments Section of your manuscript:**

**“This work was supported by the Wando Marine Healing Blue Zone Creation Project funded by the Presidential Committee for Balanced National Development in South Korea.”**

**We note that you have provided funding information that is not currently declared in your Funding Statement. However, funding information should not appear in the Acknowledgments section or other areas of your manuscript. We will only publish funding information present in the Funding Statement section of the online submission form.**

**Please remove any funding-related text from the manuscript and let us know how you would like to update your Funding Statement.**

**Please include your amended statements within your cover letter; we will change the online submission form on your behalf.**

We deleted any funding-related text from the manuscript.

And our “Funding Statement” is the same in the submitted manuscript as follows

: “This work was supported by the Wando Marine Healing Blue Zone Creation Project funded by the Presidential Committee for Balanced National Development in South Korea”

**5. We note that you have stated that you will provide repository information for your data at acceptance. Should your manuscript be accepted for publication, we will hold it until you provide the relevant accession numbers or DOIs necessary to access your data. If you wish to make changes to your Data Availability statement, please describe these changes in your cover letter and we will update your Data Availability statement to reflect the information you provide.**

We created a data accessible DOI for data sharing.

DOI : https://doi.org/10.6084/m9.figshare.21904017.v1

**6. We note that you have included the phrase “data not shown” in your manuscript. Unfortunately, this does not meet our data sharing requirements. PLOS does not permit references to inaccessible data. We require that authors provide all relevant data within the paper, Supporting Information files, or in an acceptable, public repository. Please add a citation to support this phrase or upload the data that corresponds with these findings to a stable repository (such as Figshare or Dryad) and provide and URLs, DOIs, or accession numbers that may be used to access these data. Or, if the data are not a core part of the research being presented in your study, we ask that you remove the phrase that refers to these data.**

We deleted "data not shown" written in our manuscript and we added the tables S10 and S11 to show the data previously "not shown data".

Thank you to the reviewers who carefully reviewed and to the editor who kindly requested corrections.

We look forward to hearing from you.

**Seoeun Lee**

Researcher of Lab of Environment and Health

Tel: +82 2-2286-1348

Mailing address: ANAM-DONG 5-GA, SEONGBUK-GU, SEOUL, 136-701

Email: leese1105@korea.ac.kr
