# Supplementary material for: Changes in cortisol awakening responses (CAR) in menopausal women through short-term marine healing retreat program with specific factors affecting each CAR index
Source: PLoS One. 2023 Apr 19;18(4):e0284627. doi: 10.1371/journal.pone.0284627 (PMC10115294; doi:10.1371/journal.pone.0284627)
Supplement: Attachment — Submitted filename: 230323_Response to reviewers.docx [file pone.0284627.s014.docx]

**March 23, 2023**

**TO**: Juan-Luis Castillo-Navarrete, Ph.D

**E-mail**: [em@editorialmanager.com](mailto:em@editorialmanager.com)

**Subject** : PLOS ONE - Manuscript number PONE-D-22-22264R1

**Title:** Changes in cortisol awakening responses (CAR) in menopausal women through short-term marine healing retreat program with specific factors affecting each CAR index

We really appreciate the comments of the editor and reviewers and found that they helped to improve our paper. We have revised the submitted revised manuscript again with responded to the advices of the editor and the reviewers. And the responses to each comment are as follows;

**Responses to academic editor’s comments:** The academic editor’s advice has been bold letters and our response follows. In addition, the contents reflected in the manuscript were written in red.

**(1) No mention is made in the methodology about the measurement of TG and BDNF, even more so considering the difficulties involved in measuring the latter, therefore:**

We appreciate the academic editor 's thoughtful comments. Our responses are listed below in order.

**(1.1) How was peripheral BDNF measured? Serum? Plasma? Platelets?**

We appreciate your comments.

Peripheral BDNF was measured in serum. We added the subheading about the blood sampling and analysis in the Materials and Methods.

[Materials and Methods] (7p, 128L~130L)

8. Blood sampling and analysis

Triglyceride (TG) and Brain-derived neurotrophic factor (BDNF) were assessed by measuring serum TG or BDNF levels of blood samples.

**(1.2) What were the conditions of peripheral blood sampling (a simple forced aspiration at the time of peripheral blood sampling is sufficient to degranulate platelets and thus increase "plasma" BDNF levels) (**[**https://doi.org/10.1016/j.neurobiolaging.2004.03.002**](https://doi.org/10.1016/j.neurobiolaging.2004.03.002)**).**

We appreciate your thoughtful comments.

Blood samples were collected from each participant in the fasting state on the morning of the first and last days of the program using SST tube. Simple forced aspiration during blood collection can degranulate platelets and increase "plasma" BDNF levels. However, in our study, BDNF was analyzed in serum, not plasma. Therefore, simple forced aspiration method in our study may not change the serum BDNF levels like plasma BDNF levels. In addition, we collected blood while maintaining the same conditions before and after the program, and minimized the error of BDNF measurement by comparatively analyzing BDNF levels before and after the program in the same subjects with the same procedures.

We wrote the blood collection information by adding it to the Materials and Methods.

[Materials and Methods] (7p, 129L~131L)

Triglyceride (TG) and Brain-derived neurotrophic factor (BDNF) were assessed by measuring serum TG or BDNF levels of blood samples. Blood samples were collected from each participant using the SST tube in the fasting state on the morning of the first and last days of the program.

**(1.3) Was anticoagulant used to receive the peripheral blood sample? Which one?**

We greatly appreciate the comments.

Blood was collected using the SST tube (Serum separator tube). It is a tube for serum separation and does not contain any anticoagulants. We wrote the blood collection tube information by adding it to the Materials and Methods.

[Materials and Methods] (7p, 130L~131L)

Blood samples were collected from each participant using the SST tube in the fasting state on the morning of the first and last days of the program.

**(1.4) How long were the collection tubes left for before centrifugation, how long were they centrifuged, at what speed?**

We really appreciate the comments.

In our study, blood samples were mixed up and down the tube 6 times immediately after blood collection, and then left at room temperature for 30 minutes. And centrifuged at 3000 rpm for 10 minutes. We wrote this information by adding it to the Materials and Methods.

[Materials and Methods] (7p, 131L~133L)

Immediately after blood collection, the tube was mixed up and down 6 times, and then left standing at room temperature for 30 minutes. The tube was centrifuged at 3000 rpm for 10 minutes.

**(1.5) What technique was used to measure BDNF? ELISA? If so, does it adequately discriminate mature BDNF from proBDNF (**[**http://dx.doi.org/10.1038/srep17989**](http://dx.doi.org/10.1038/srep17989)**)?**

We appreciate your thoughtful comments.

In our study, Total BDNF Quantikine ELISA Kit (R&D Systems, USA) was used for BDNF measurement. It does not differentiate between mature BDNF and pro BDNA. However, it was reported in the paper mentioned above that this ELISA kit we used for analysis showed a slight reactivity to pro BDNF and the results by the kit could be regarded as the level of mature BDNF. Although our study has a limitation that it could not accurately distinguish between pro BDNF and mature BDNF, it seems that our results more likely reflect the levels of mature BDNF based on the performance of ELISA kit analysis report. We wrote this content by adding it to the Materials and Methods and the Discussion.

[Materials and Methods] (7p, 136L~137L)

BDNF (pg/ml) concentrations was measured using Total BDNF Quantikine ELISA Kit (R&D Systems, USA) and SpectraMax 190 Microplate Reader (Molecular Devices, USA).

[Discussion] (13p, 270L~273L)

And the BDNF assay method used in our study mainly reflects mature BDNF in serum based on the report about the performance of ELISA kit analysis which showed a slight reactivity to pro BDNF (54). Therefore, our results suggested that the BDNF changes through exercise program showed the consumption of mature BDNF.

**(1.6) How were TGs determined? methodology? sampling conditions?**

We really appreciate the comments. And we are sorry for our mistakes not filling out the information on TG and BDNF analysis including blood sampling.

TG was also measured in serum collected under the same blood collection conditions as BDNF, and the concentration was analyzed using Enzymatic assay (ADVIA® Chemistry XPT System; Siemens, Munich, Germany). We wrote this information by adding it to the Materials and Methods.

[Materials and Methods] (7p, 128L~137L)

8. Blood sampling and analysis

Triglyceride (TG) and Brain-derived neurotrophic factor (BDNF) were assessed by measuring serum TG or BDNF levels of blood samples. Blood samples were collected from each participant using the SST tube in the fasting state on the morning of the first and last days of the program. Immediately after blood collection, the tube was mixed up and down 6 times, and then left standing at room temperature for 30 minutes. The tube was centrifuged at 3000 rpm for 10 minutes. Blood samples were sent to the LabGenomics Laboratory (Korea), stored at -80 degrees until analysis. TG (mg/dL) concentrations was measured using Enzymatic assay (ADVIA® Chemistry XPT System; Siemens, Munich, Germany). BDNF (pg/ml) concentrations was measured using Total BDNF Quantikine ELISA Kit (R&D Systems, USA) and SpectraMax 190 Microplate Reader (Molecular Devices, USA).

**(2) It is hypothesised that the exercise programe would reduce BDNF levels, how is this explained? Even more so, when there is ample literature reporting increased BDNF levels as a result of physical exercise and cognitive training (**[**https://doi.org/10.1038/s41380-019-0639-2**](https://doi.org/10.1038/s41380-019-0639-2)**;** [**https://doi.org/10.3390/ijms22168814**](https://doi.org/10.3390/ijms22168814)**).**

We appreciate your thoughtful comments.

We also previously identified literature reporting increased BDNF levels as a result of physical exercise and cognitive training. Long-term exercise interventions ranging from 4 to 48 weeks have been included in a number of related studies. And in most of those studies, blood levels of BDNF were increased. However, overweight and obese subjects participated a 12-week aerobic exercise showed 20% decrease of serum BDNF. We cited this paper (ref. 46) in the Discussion at line 254 already. And our study confirmed changes in blood BDNF due to the effects of short-term exercise intervention in less than a week. To our knowledge, few studies have reported to measure the changes in BDNF through short-term exercise interventions like our study. In addition, a study reporting a decrease in serum BDNF after 3 months of long-term exercise suggests the possibility that BDNF is consumed and reduced to protect mechanical stress caused by exercise and repair of peripheral nerves or tissues (<https://doi.org/10.1016/j.neulet.2008.03.057>). By a similar mechanism, our results suggest that short-term exercise interventions can use serum BDNF for exercise-induced oxidative stress protection and damage repair. We wrote this by adding it to the Discussion.

[Discussion] (13p, 257L~266L)

While physical activity promotes health benefits, strenuous or prolonged exercise increases the production of reactive oxygen species in the blood and skeletal muscles (48). Previous studies have reported that the circulating BDNF can act as a protective agent against oxidative damage; as oxidative stress increases and the BDNF levels decrease (49, 50). In addition, a study reporting a decrease in serum BDNF after 3 months of long-term exercise suggests the possibility that BDNF is reduced by exercise-induced mechanical stress and consumed to promote repair of peripheral nerves or tissues (51). Our study has the limitation of not directly investigating changes in oxidative stress. However, through a mechanism similar to previous studies, our results suggest that serum BDNF has the potential to be used for protection and damage repair against exercise-induced oxidative stress through short-term exercise interventions.

**(3) In the supplementary tables the units of TG and BDNF are not indicated.**

We greatly appreciate the comments. The supplementary table has been modified to indicate the units of TG and BDNF.

[supplementary] (Table S12)

**Table S12.** Differences in the changes of TG and BDNF depending on BMI groups through the marine healing program

| **BMI group** | **n** | **Change of TG**  **(mg/dL)** | **p** | **Change of BDNF**  **(pg/ml)** | **P** |
| --- | --- | --- | --- | --- | --- |
| Normal & Overweight (<25) | 27 | -42.9 ± 31.7 | 0.007^**^ | -6134 ± 9958 | 0.04^*^ |
| Obese (25~) | 24 | -84 ± 69.5 |  | -13161 ± 13322 |  |

Each bar represents the mean ± SD. p-values were obtained by student t-test. *p-value<0.05;**p < 0.01. TG : triglyceride; BDNF : brain-derived neurotrophic factor.

**(4) The above points are relevant to clarify, even more so when peripheral BDNF levels have been proposed as a way to assess the integrity of the HPA axis, given its inverse relationship with cortisol levels (**[**http://dx.doi.org/10.1038/s41386-019-0391-y**](http://dx.doi.org/10.1038/s41386-019-0391-y)**;** [**https://doi.org/10.3109/07853890.2015.1131327**](https://doi.org/10.3109/07853890.2015.1131327)**).**

We appreciate your thoughtful comments with the information that there are associations among BDNF, stress and HPA axis activity. Including the papers cited above, several studies have reported that BDNF is increased in response to acute stress, and BDNF is decreased in people with chronic stress or mental illness. However, subjects with psychological disorders were excluded in our study and we found no significant changes in stress markers (Table 6). In addition, all subjects performed the same schedule through the marine retreat program, so the stress factor was somewhat controlled. And the decrease of BDNF was found with the increase of CAR in obese group. However, this inverse association may not directly related to the change of stress because of no significant change of stress markers. Therefore, we suggest that our findings are independent of stressors and could be interpreted as exercise-induced physiological changes to BDNF levels or CAR curves in separate ways. We wrote this by adding it to the Discussion.

[Discussion] (13p, 267L~271L)

In addition, several studies have reported decreased BDNF in patients with chronic stress or insomnia (52, 53). However, our study showed no significant changes in stress markers (Table 6) with increase of CAR and decrease of BDNF. These findings suggested that the effect of exercise, independent of the stress factor, had a physiological effect to BDNF levels or CAR curves in separate ways.

**Responses to reviewer’s comments:** The reviewer’s advice has been bold letters and our response follows. In addition, the contents reflected in the manuscript were written in red.

**Reviewer #1:**

(1) **I got to read the first version of the MS. I must admit that this second version of the manuscript has been greatly improved. However, it still needs more care in the introduction/discussion/as well as in the abstract. There are many inaccuracies, with sentences left there in inappropriate places: results are still presented in the introduction or extensions of the research on absolutely different topics are hypothesized.**

Thank you very much for your thoughtful comments. We have corrected the details of the manuscript according to your advice. We deleted repetitive expressions in the Abstract and corrected the content as follows.

[Abstract] (2p, 21L~23L, 31L~33L)

However, it is unclear which physiological phenomenon each index reflects. ~~In addition, although the factors affecting each index, such as stress, circadian rhythm, sleep, and obesity, are being examined, further studies are needed.~~

The obese group also showed significantly decreased serum triglyceride and BDNF (brain-derived neurotrophic factor) levels compared to the low BMI group. ~~suggesting that the marine retreat-based healing program was more effective in the obese group.~~

We changed "parameter" to "index" in abstract and reason of change was described in the response related to clarifying the CAR types below.

[Abstract] (2p, 21L, 27L~28)

However, it is unclear which physiological phenomenon each index reflects.

The baseline CAR indices showed that the AVE and AUCg were significantly higher in the high sleep efficiency group than in the low sleep efficiency group.

We supplemented the sentences in the Abstract.

[Abstract] (2p, 21L, 23L~25)

This study investigated the factors, such as stress, circadian rhythm, sleep, and obesity, affecting the CAR through a marine retreat-based healing program in which the anticipated stress of the participants could be controlled to some degree.

We have deleted and corrected the content of the results remaining in the Introduction.

[Introduction] (4p, 76L~78L)

healing retreat program, including beach yoga and Nordic walking. ~~We found that CAR was associated with sleep efficiency before the program, and a change in CAR was associated with obesity throughout the exercise retreat program.~~

We corrected sentences whose meaning was not clear and supplemented the sentences in the Results.

[Result] (9p, 169L~172L)

Each CAR index was analyzed by grouping sleep-associated variables, including melatonin, sleep efficiency, and circadian rhythm variables. And the circadian rhythm was measured by the amplitude and phase of the cosine curve of heart rate. And there was no significant difference in sleep efficiency by age group (Table S1). Each CAR index showed variable results by sleep efficiency factor.

We have deleted sentences presented as extending to other topics and corrected the sentences in the discussion.

[Discussion] (13p, 253L~257L)

Aerobic exercises reduce the TGs in adults who are overweight or have obesity (45), and exercising and dietary control have been shown to reduce BDNF in women with obesity (46). ~~In addition, a study has reported that the recognition of bodyweight-associated stigma increases oxidative stress and activates the HPA axis, which affects the CAR (47).~~

[Discussion] (15p, 303L~305L)

Further studies with larger populations are needed to validate the factors such as sleep efficiency, obesity and aging affecting each CAR index.~~, anticipating new information in the management and treatment of sleep disorders and obesity by using CAR parameters.~~

We corrected sentences whose meaning was not clear and supplemented the contents in the Discussion. And we also revised or added some sentences in the Discussion according to comments of the academic editor.

[Discussion] (11p, 214L~216L)

These results are consistent with Anderson and Wideman’s report (41), in which each index of CAR may reflect different physiological phenomena.

[Discussion] (13p, 259L~266L)

Previous studies have reported that the circulating BDNF can act as a protective agent against oxidative damage; as oxidative stress increases and the BDNF levels decrease (49, 50). In addition, a study reporting a decrease in serum BDNF after 3 months of long-term exercise suggests the possibility that BDNF is reduced by exercise-induced mechanical stress and consumed to promote repair of peripheral nerves or tissues (51). Our study has the limitation of not directly investigating changes in oxidative stress. However, through a mechanism similar to previous studies, our results suggest that serum BDNF has the potential to be used for protection and damage repair against exercise-induced oxidative stress through short-term exercise interventions.

[Discussion] (13p, 267L~274L)

In addition, several studies have reported decreased BDNF in patients with chronic stress or insomnia (52, 53). However, our study showed no significant changes in stress markers (Table 6) with increase of CAR and decrease of BDNF. These findings suggested that the effect of exercise, independent of the stress factor, had a physiological effect to BDNF levels or CAR curves in separate ways. And the BDNF assay method used in our study mainly reflects mature BDNF in serum based on the report about the performance of ELISA kit analysis which showed a slight reactivity to pro BDNF (54). Therefore, our results suggested that the BDNF changes through exercise program showed the consumption of mature BDNF.

[Discussion] (14p, 286L~287L)

Therefore, further comprehensive studies are needed to evaluate the significant factors for each CAR index changes to ~

(2) **I am referring to the possible involvement of the mechanisms underlying oxidative stress in the effects induced by marine living on CAR. Why not bring up the stimulating effect of the marine environment on the thyroid?**

We greatly appreciate the comments. As you suggested, the thyroid stimulating effect of the marine environment is a very important issue. We also found the changes in thyroid hormone levels through marine retreat program. And we also found the associations between changes in thyroid hormone levels and changes of HRV values. So, we recently published a separate paper on this issue (Title : Effect of the Marine Exercise Retreat Program on Thyroid-Related Hormones in Middle-Aged Euthyroid Women, <https://doi.org/10.3390/ijerph20021542>). In preparing the above paper, there was no correlation between thyroid hormone and CAR, so this study did not include the effect on the thyroid hormone caused by the exposure to marine environment.

(3) **I want to clarify that, in my opinion, you have not studied three different types of CAR but rather have used different ways of mathematically expressing the physiological increase in cortisol, which is evident in the first hour after waking up. It is up to the authors to decide which method is best adapted to the proposed protocol and thus make it easier to read the work.**

We appreciate your comments. We were impressed by your comment “different ways of mathematically expressing the physiological increase in cortisol”. In fact, to evaluate CAR, previous studies developed a CAR index through various mathematical calculations (<https://doi.org/10.1016/j.psyneuen.2018.05.034>). And we used the three most commonly used indices in CAR studies. We recognized that the expression “parameter” may not be easily understandable by readers. Therefore, we changed all the word ‘parameter’ to the word ‘index’ according to previous study. We would appreciate it if you could check the manuscript for the corrected words.

[Title] (1p, 1L~2L)

Changes in cortisol awakening responses (CAR) in menopausal women through short-term marine healing retreat program with specific factors affecting each CAR index

[Result] (9p, 164L~165L)

All the CAR indices showed a decreasing trend among the older age groups. The AUCi for the CAR indices was significantly~~

(4) **In addition, even before talking about further studies, the authors should still apply themselves to make this work intelligible and acceptable according to the standards set by PlosOne.**

We really appreciate your advice. Your comments have been very helpful in developing our study. Therefore, we did our best to revise the manuscript to meet the reviewer's advice and PLOS ONE's requirements.

**Reviewer #2:**

**I appreciate the effort in restructuring the manuscript to respond to the reviewers' comments. The structure of the new manuscript satisfies the requirements sent by the reviewers, however I suggest two minor revision:**

(1) **In p9 L181 include "normal" to compare with high:...more in the group with a "normal" waist-to-hip ratio (WHR) than...**

We greatly appreciate the comments. We have corrected that sentence according to your advice.

[Result] (9-10p, 182L~184L)

The results showed that AUCi increased significantly more in the group with a normal waist-to-hip ratio (WHR) than in the group with a high WHR (p=0.02).

(2) **Considering the adjustments made in the manuscript, it seems appropriate to include in the results a brief description about the supplementary tables S10 and S11, in order to guide the reader in the subsequent discussion of the scopes of the study.**

Thank you very much for your thoughtful comments. The descriptions of the tables have been modified to be included in the results. Please note that the number of tables has changed according to the order in which they are written in the manuscript.

[Result] (9p, 170L~172L)

And there was no significant difference in sleep efficiency by age group (Table S1).

[Result] (10p, 197L~201L)

multivariate regression analysis showed that BMI was the factor that significantly affected changes in AVE and AUCg during the program and not sleep efficiency (Tables S8 and S9). No factor that significantly influenced AUCi was found (Table S10). In addition, it was confirmed that there was no significant difference in exercise intensity according to the BMI group (Table S11).

We thank the academic editor and reviewers for their careful review.

We look forward to hearing from you.

**Seoeun Lee**

Researcher of Lab of Environment and Health

Tel: +82 2-2286-1348

Mailing address: ANAM-DONG 5-GA, SEONGBUK-GU, SEOUL, 136-701

Email: leese1105@korea.ac.kr
